# Machine Learning Interatomic Potentials and Long-Range Physics

**DOI:** 10.1021/acs.jpca.2c06778

**Published:** 2023-02-21

**Authors:** Dylan M. Anstine, Olexandr Isayev

**Affiliations:** Department of Chemistry, Mellon College of Science, Carnegie Mellon University, Pittsburgh, Pennsylvania 15213, United States

## Abstract

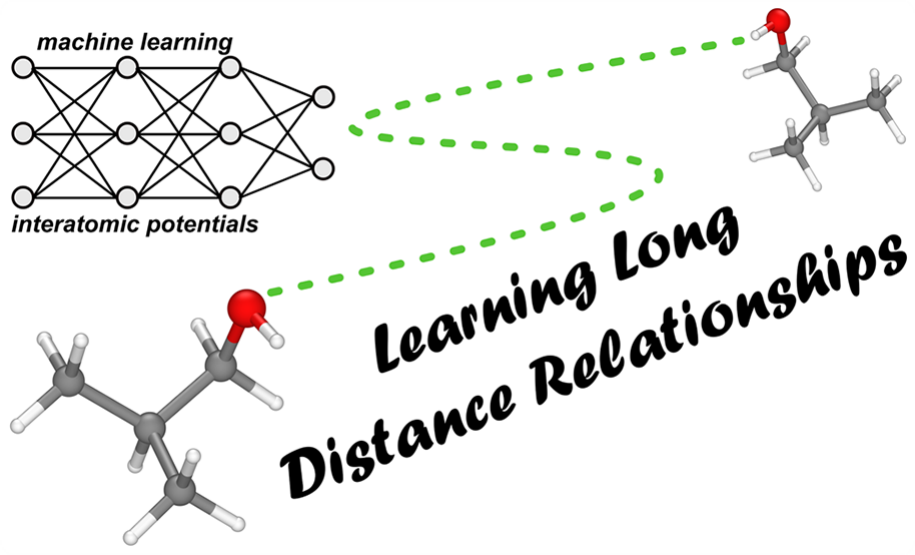

Advances in machine learned interatomic potentials (MLIPs), such as those using neural networks, have resulted in short-range models that can infer interaction energies with near ab initio accuracy and orders of magnitude reduced computational cost. For many atom systems, including macromolecules, biomolecules, and condensed matter, model accuracy can become reliant on the description of short- and long-range physical interactions. The latter terms can be difficult to incorporate into an MLIP framework. Recent research has produced numerous models with considerations for nonlocal electrostatic and dispersion interactions, leading to a large range of applications that can be addressed using MLIPs. In light of this, we present a Perspective focused on key methodologies and models being used where the presence of nonlocal physics and chemistry are crucial for describing system properties. The strategies covered include MLIPs augmented with dispersion corrections, electrostatics calculated with charges predicted from atomic environment descriptors, the use of self-consistency and message passing iterations to propagated nonlocal system information, and charges obtained via equilibration schemes. We aim to provide a pointed discussion to support the development of machine learning-based interatomic potentials for systems where contributions from only nearsighted terms are deficient.

## 1 Introduction

For the last several decades ab initio molecular simulations have been instrumental in overcoming challenges faced by the chemical and materials sciences.^1^ These methods have a foundation in the theories of quantum mechanics (QM) and offer computational scientists a means of understanding the atomistic- and electronic-level details governing material and molecule behavior. The value of ab initio molecular simulations is proven, and increasing computational power is furthering their widespread use, e.g., the development of exascale computing.^2^ Despite this progress, many systems remain too large, and many topics require a number of simulations too great to be investigated solely by QM calculations. The rise of data-driven techniques, particularly simulations performed with machine learned interatomic potentials, has demonstrated the possibility to explore these otherwise computationally demanding areas without sacrificing ab initio accuracy.^3^

The utility of particle-based molecular and materials simulations is connected with generating an accurate potential energy surface (PES) representation, which is a landscape that underpins reactivity, phase stability, and other observable properties. Arguably, a “holy grail” for computational scientists is to efficiently sample large numbers of readily available and exceptionally accurate PESs. High-quality PESs are typically obtained using QM methods, but there is an appreciable cost associated with the level of theory used to approximate solutions to the Schrödinger equation.^4^ Considering an example of a multielectron (N_elec_) system, the number of arithmetic operations for a QM calculation could scale from order O(N_elec_^3^) to order O(N_elec_^7^) depending on the method needed to achieve the desired accuracy.^5^ In cases where a system has more than 10^2^ atoms, the computational requirement of QM methods is dissuading, and computational chemists and materials scientists are relegated to using lower-dimensional representations that trade PES details for efficiency. Fortunately, this accuracy–efficiency trade-off, classically thought of as pervasive and unavoidable, is beginning to be overcome by the rapid expansion of machine learned interatomic potentials (MLIPs).^6−8^ MLIPs can be constructed using numerous techniques, such as neural networks, reinforcement learning, or kernel methods. We choose to leave discussion on the subtleties of different approaches to available reviews^9−12^ and, instead, highlight herein the specifics regarding MLIPs with long-range interaction strategies.

**Figure 1:**
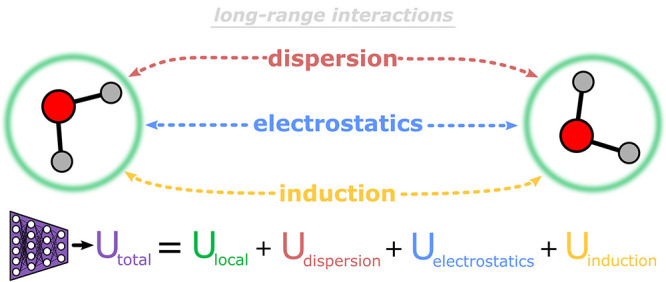
Summary of the general energetic contributions composing the total potential energy (*U*_total_) of a system. *U*_local_ refers to the short-range system energies and is typically inferred using a machine learning model trained on local features. Dispersion corrections, electrostatics, and induction are collectively referred to as the long-range interaction energy contributions.

The overarching concept of MLIPs is for a model to *learn* the relationship between a set of atomic/molecular features and accurate training data. Reference energy and forces can be obtained using high-throughput QM calculations, where the goal of the resulting MLIP model is to achieve near ab initio accuracy with orders of magnitude lower computational cost. Following successful training and validation, an MLIP can infer the interaction energies of systems with feature space representations near the training data. The inference process can be referred to as interpolation and extrapolation; however, the distinction between these terms may lack clarity in high-dimensional model spaces.^13^ Many MLIPs reported thus far choose descriptors of local atomic environments for model training. Relying on local features as MLIP input vectors can introduce complications for simulating extended systems with relevant long-range interactions,^14^ see Figure 1. Heterogenous bulk phases,^15^ dispersion,^16^ hydrogen bonding,^17^ and extended charge transfer^18^ are a few types of phenomena that require careful consideration when constructing an MLIP model. Therefore, while the idea of MLIPs may present itself as straightforward, complexities quickly arise depending on the physicochemical behavior governing the systems of interest.

In our experience, one objection to adopting MLIPs is a lack of demonstrated ability to capture physics and chemistry beyond short-range cutoffs (e.g., 5 Å). Recent progress has produced various MLIP models with simple to sophisticated treatments of long-range interactions,^19^ and such an objection is beginning to lose its basis. Despite such developments, demonstrations that these MLIPs can be used to address chemical and materials science challenges are lacking. Future efforts are needed to validate that interaction mechanisms used by modern MLIPs can successfully reproduce relevant long-range physics and chemistry in an application or experimental setting. Thus, this Perspective provides a timely and focused discussion on the current state of developing MLIPs that include long-range interactions. It should be noted that our use of the term *long-range* covers the energetic contributions and structural features exhibited on length scales beyond truncated local atomic environments (see Section 2), which we will refer to as the short-range or nearsighted potentials. A brief background on short-range potentials is given to frame the challenge of including long-range interactions with MLIPs. Intricacies of machine learning practices and an extensive description of the evolving machine learning landscape in the chemical sciences is outside the topic of this Perspective, and readers are directed to a number of reviews covering these details.^6,19−22^ Overall, we discuss models and methods that can be applied to the two major long-range interaction components: dispersions and electrostatics, where the latter has experienced significant methodological development.

## 2 Background

Prior to expanding on long-range physical interactions, the key concepts used in developing intrinsically short-range MLIPs are summarized. Our focus is on neural network potentials (NNPs) that utilize an atomic environment vector (AEV) input representation that shares Behler-Parrinello and ANI-like functional forms.^23,24^ This input representation is chosen for the general illustration of the issues that nearsighted potentials face. Several other input representations exist,^25−27^ and they can also suffer from similar deficiencies. These include spectral neighbor analysis,^28^ smooth overlap of atomic positions,^29^ and invariant polynomials,^30^ to name a few. Regardless, the local interaction energy (*U*_local,MLIP_) of an N-particle system can be described as a sum of each particle’s potential energy contribution (*U*_*i*,MLIP_).
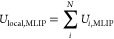
1

The magnitude of *U*_*i*,MLIP_ is a function of the environment that particle *i* is present in; i.e., the energy is defined by the collection of neighboring particles and their relative positions. NNPs utilize eq 1 by computing particle interaction energies with machine learned predictions based on atomic environment vectors as inputs. An AEV is constructed by defining a suitable cutoff function for pairwise neighbor distances (*r*_*ij*_) that smoothly approaches a value of zero at a defined cutoff (*R*_c_). ANI potentials utilize a piecewise cosine cutoff function (*f*_c_) with the following form.
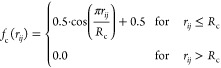
2

The cutoff function is a main component to calculate atom-centered symmetry functions (G), which serve as invariant input vectors for machine learned interatomic potentials. Imposing a cutoff is a key topic because it enforces that AEVs are short-range by design, and therefore, the resultant neural network potential is short-range without additional considerations. The AEV, defining an atom’s local environment, can be composed of several two-body radial (*g*_*m,i*_^R^) and three-body angular symmetry (*g*_*m,i*_^A^) functions.
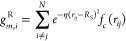
3
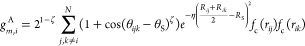
4

In these equations, the values *R*_S_ and θ_S_ are shifting factors used to probe specific regions of particle *i*’s local radial and angular environments, respectively. η and ζ control the amount of the atomic environment observed by each probe. A set of shifting and width parameters is denoted by the subscript *m*, whose systematic variation allows one to include chemical features across the local atomic environment up to the cutoff. The collection of *G* values formed across sets *m* compose the AEV input representation used by machine learning models. Overall, *R*_S_, θ_S_, η, and ζ values are selected to yield an AEV resolution that distinguishes between diverse systems without unnecessary *G* calculations. The development of eqs 3 and 4 from the cosine cutoff function introduces a challenge for accurately representing a PES, namely, AEVs built from these functions produce machine learned (ML) models blind to long-range structure and physical phenomena.^31^

Depending on the construction of the AEV input representations, an ML potential model can be trained to infer atomic interaction energies and forces. To provide a concise overview, we restrict our following discussion mainly to deep NN models; however, similar considerations apply for alternative ML approaches, for example, Gaussian-approximation potentials (GAPs).^32^ In multilayer NN models, the input representation and output energy are the physically relevant quantities. The intermediate layers consist of parameters that are optimized during training to provide the nonlinear transformation of the input representation needed for an accurate inference of energy and forces.

## 3 MLIPs with Long-Range Interactions

Long-range interactions in extended chemical systems contribute to numerous physical phenomena: e.g., thermodynamic phase behavior,^31^ variations in conformer geometry,^33^ permeation rates of molecules through porous materials,^34^ protein structure and dynamics,^35^ self-assembly or directed assembly of macromolecular complexes,^36^ and interfacial properties.^37^ It could be suggested that interactions that occur over longer length scales can be directly predicted with an MLIP model by increasing the cutoff radius used by the atom-centered spherical symmetry functions (or equivalent local atomic descriptor). To a certain extent, it is possible to include longer-range contributions with model components specifically trained to reproduce intermolecular interactions; for instance, AP-NET utilizes 8 Å cutoff atom-pair symmetry functions for evaluating monomer–monomer interaction energies.^38^ There is an eventual pitfall for increasing the AEV cutoff: namely, the number of descriptor calculations increases, which yields larger compute requirements to capture physical interactions that slowly decay with separation distance. The learning task can also become more challenging due to the fact that the chemical configuration space grows with larger cutoff radii, thus, the issue of sufficient sampling compounds.

**Figure 2:**
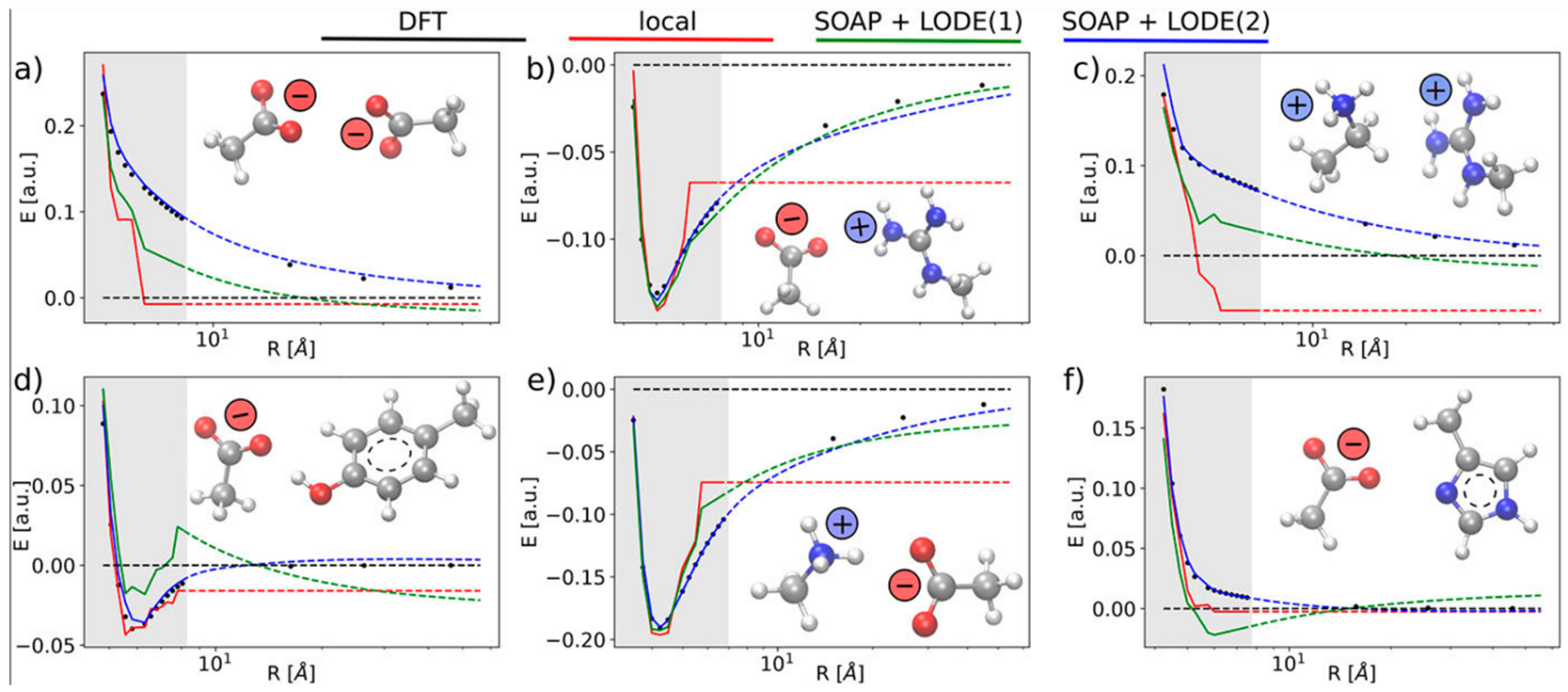
Example of long-range interaction relevance using charged dimer dissociation curves. Non-negligible interaction energies are captured with separation distances up to tens of nanometers. Black dots are DFT reference calculations, red lines are MLIPs with only local descriptors, and green/blue lines are nonlocal MLIP models presented by Grisafi and Ceriotti.^41^ Reprinted with permission from ref (41). Copyright 2019, American Chemical Society.

Distance scaling relationships of long-range interactions vary depending on the systems studied and the physical phenomena considered. Most often applied in classical empirical force fields are Coulomb and Lennard-Jones potentials, displaying *r*_*ij*_^–1^ and *r*_*ij*_^–6^. Beyond these common functional forms, polarization contributions display *r*_*ij*_^–4^ distance scaling for the leading interaction term (monopole-dipole) of the induction energy.^39^ Even further, work by Ambrosetti demonstrated that the power-law scaling of van der Waals interactions can range between ca. −2 and −5 depending on the type of nanostructure and separation distance, which is a result of wavelike fluctuations in charge density.^40^ This list of slowly decaying distance relationships is not exhaustive, but it emphasizes that MLIP developers need to carefully consider the length scales of chemical and physical interactions underpinning the target application space. As a specific example, the dissociation curves of charged dimers are cases where non-negligible long-range energy contributions are observed up to tens of angströms, see Figure 2.

Most MLIPs can be classified according to three main strategies when considering their treatment of long-range interactions and nonlocal phenomena: (1) neglect their contribution and only include short-range features, (2) augment MLIPs with standard long-range functional forms, e.g., Coulomb’s law, where parameters have local environment dependency, and (3) MLIPs with long-range interactions that are sensitive to global system characteristics. The choice to adopt one of these three strategies should be physically motivated. As an example, systems with a narrow range of elements and short screening distances can oftentimes be simulated using strategy 1 with minimal loss of accuracy, which has been demonstrated across many studies.^23,42−45^ In these cases, the adoption of strategy 2 or 3 results in additional computational expense and training complications without appreciable accuracy gain for the target application. On the contrary, employing strategy 1 for systems displaying phenomena such as long-range polarization and electrostatics will likely lead to poor predictions. The concept of choosing an appropriate model design for a given system is well-known in traditional molecular simulations and extends to those being performed with MLIPs.

### 3.1 Dispersion Corrections

van der Waals forces are a ubiquitous type of atomic interaction that originate from fluctuations in electron density distributions.^46^ They can be partitioned into short-range repulsion terms and long-range attraction; the latter being referred to as the dispersion interaction. It is not necessary to expand on the inclusion of short-range repulsions in our MLIP discussion because they can be learned by a nearsighted model or explicitly added using a collection of empirical terms, such as the Ziegler-Biersack-Littmark (ZBL) potential.^47,48^ For small isolated systems, which define the application space of many reported MLIPs, the contribution of dispersion interactions can be minimal, and as a result, their neglect or inclusion may not affect inference accuracy. However, the collective strength of dispersion forces in larger systems is often non-negligible, and they can be a significant factor in defining properties such as polymer cohesive energy density^49^ and organic molecular complex stability,^50^ to name a few. Therefore, it is worthwhile to expand on the treatment of long-range dispersion interactions in the design of an MLIP.

One level of consideration for dispersion interactions is in the construction of the reference training data. MLIP model developers are often guided by the goal of obtaining density functional theory (DFT)-quality accuracy with near empirical potential computational cost. This often equates to applying dispersion correction strategies^51^ to DFT calculations that use, for instance, generalized gradient approximation density functionals,^52^ or one can choose to neglect dispersion correction in the reference data and include them explicitly as part of the MLIP model. As an example, the ANI family of MLIPs is based on locally defined symmetry functions (see Section 2), yet dispersion corrections are applied to the entire system *ad hoc*, regardless of AEV cutoff, to expand the interaction range of the otherwise inherently nearsighted models. As a counterexample, Morawietz et al.^53^ trained a set of models, also based on locally defined symmetry functions, directly to dispersion-corrected reference data to obtain good agreement with the density maximum of bulk water studied as a function of temperature. The key distinction between these two examples is whether dispersion correction is applied “on-the-fly” during inference or learned implicitly by the MLIP. The latter strategy can be more computationally efficient; however, it will ultimately lead to incorrect predictions for systems where dispersion contributions beyond the local AEVs cannot be neglected. The MLIP training data of Morawietz et al. consisted of DFT calculations augmented with the D3 dispersion correction scheme from the 2010 landmark work of Grimme and co-worker.^54^ D3-corrected DFT calculations are a frequently used tool for construction of MLIP reference training data for organic systems; see eq 5 for the 2-body dispersion correction functional form
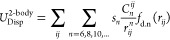
5where *s*_*n*_, *C*_*n*_^*ij*^, and *f*_d.n_ represent the scaling factors, dispersion coefficients, and damping functions, respectively. Despite the popularity, it is worth commenting that dispersion-corrected DFT is an active field of research and defaulting to D3 corrections could incur error depending on the chemical diversity and/or system size reflected in the target application space.^55,56^ Moreover, the importance of many-body dispersion effects is another consideration, which can result in non-negligible interaction energy contributions with increasing system size. As an example, Tkatchenko et al. used crystalline benzene as a model system to demonstrate that pairwise Tkatchenko-Scheffler dispersion corrections overestimated the crystal cohesive energies and that a many-body dispersion (MBD) correction based on coupled and uncoupled quantum harmonic oscillators substantially corrected this error.^57^ In cases where the D3 method of Grimme is insufficient, one could seek to improve the interaction energies by including a 3-body dispersion term (C_9_) with the Axilrod–Teller form.^58^ These two examples are select cases among many, and those looking to develop MLIPs that can be applied to systems with long-range and many-body dispersion effects are directed to the in-depth discussion provided by Hermann, DiStasios, and Tkatchenko^46^ or Grimme and co-workers.^59^

**Figure 3:**
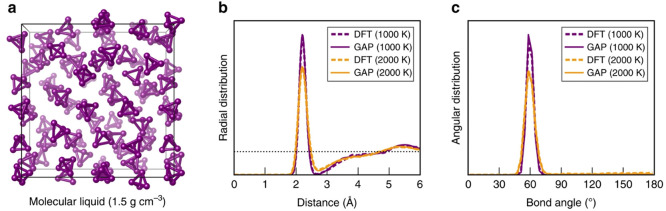
Molecular dynamics simulation results using a Gaussian approximation potential (GAP) to model the interactions of condensed-phase phosphorus. (a) Visualization of the liquid-phase phosphorus simulation cell. (b) 2-Body distance correlation function, where DFT (purple) is the reference calculation and GAP (orange) is the Gaussian approximation potential ML model. (c) 3-Body angular correlation function. Reprinted with permission from ref (60). Copyright 2020, The Authors.

For bulk systems with long screening distances, an MLIP built strictly using *U*_local_ trained to DFT-D reference data can be insufficient. This is related to the previously discussed limitation of nearsighted potentials, namely, they lack knowledge of structure or interactions beyond the local atomic environment. Instances where nonlocal structure impacts local electron density fluctuations can require dedicated terms for accurate modeling. Deringer et al. successfully implemented one solution, where an explicit two-body *r*_*ij*_^–6^-dependent dispersion interaction term is used to reasonably reproduce the structure of condensed-phase phosphorus (see Figure 3) by training on DFT data from calculations using the PBE functional and many-body dispersion correction.^60^ Condensed-phase phosphorus is challenging to simulate because of its mixture of covalent and noncovalent features; however, it could be successfully modeled using only *U*_local,MLIP_ and a two-body *r*_*ij*_^–6^-dependent dispersion term because the systems were chemically homogeneous and neutral. The concept of including an *r*_*ij*_^–6^ dispersion interaction term alongside an ML-based *U*_local,MLIP_ calculation was also used by Wen and Tadmor for multilayer graphene, which was similarly trained with PBE and many-body dispersion-corrected DFT calculations.^61^ The work by Muhli et al.^62^ utilized a different strategy, where short-range descriptors were used to predict the effective atomic Hirshfield volumes needed to employ the dispersion correction scheme of Tkatchenko and Scheffler,^63^ which enabled them to investigate the C_60_ phase diagram and associated driving forces up to 5000 K and 1000 GPa. Their model, trained on PBE reference data from DFT calculations, qualitatively reproduced a number of the carbon structure observations obtained experimentally. The successes of these two carbon MLIPs have a similar basis as the model by Deringer et al., namely, contributions from long-range electrostatics and polarization can be neglected because the systems are chemically homogeneous and treated as closed-shell. Incorporating explicit dispersion energies with parameters (or their dependencies) obtained from ML-based predictions using local structure is an approach that conceivably encourages NNP generalizability and is worth further exploration.

### 3.2 Inference of Ab Initio Point Charges

Augmenting a short-range MLIP with an explicit electrostatic energy term (*U*_es_), see eq 6, is a straightforward approach to include long-range interactions between charge sites into an ML-based model. From a quantum mechanical view of atoms, *U*_es_ is a complicated function of the electron density, and it is oftentimes computationally convenient to condense this distribution onto localized point charges. One difficulty that arises when using this strategy to account for long-range electrostatic interactions is that mapping electron density/electrostatic potentials onto point charges is an ambiguous task that lacks a unique solution. This has led to numerous electron density partitioning schemes^64−66^ that all give distinct atom-centered point charges.^67^ The consequence is that NNPs can be constructed using a variety of charge assignment strategies that vary in transferability, yielding an important consideration for interpreting simulation results.
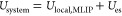
6

Some developers opt to augment their ML-based short-range potentials with Coulomb’s law and Ewald summation using static formal charges. This strategy is useful when integer charges can be rationally assigned using fundamental chemical principles; for example, this has been demonstrated in simulations of crystalline nitride materials.^68^ Electrostatic interactions based on formal charges are simple but narrow in the types of systems they can be applied to, and such a strategy is not applicable to most organic systems. Atom-centered porges of organic species are typically noninteger and exhibit variation with the atomic local environment. This originates from chemically identical molecules with unique conformations displaying differences in electron density distribution. One can address conformational-dependent partial charges by training an ML model to predict them from the AEV input representations. The system energy can be reformulated as a local interaction energy with monopole electrostatic contributions as
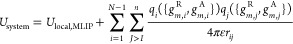
7where *q*_*i*_ and *q*_*j*_ are the charges on atoms *i* and *j* separated by a distance *r*_*ij*_, and ε is the screened dielectric constant. We are omitting dispersion and induction terms from eq 7 for clarity. It is important to note that this equation is only valid when phenomena related to electron density (re)distribution—for example, induction or multipole electrostatics—are dominated by features on a length scale less than the *U*_local,MLIP_ cutoff. Instances where this is not the case are discussed further in Section 4. Moreover, eq 7 can only be appropriately applied for systems where the dielectric screening is homogeneous. Regardless, learning conformation-dependent charges is used by several MLIPs, including PhysNet,^69^ HIP-NN,^70^ and models by Behler and co-workers.^71,72^ It is worth highlighting that charges predicted from ML models may need to be adjusted to ensure they sum to the net charge of the system. Choosing to use simple charge distribution normalization or more complex species-specific weighting schemes can affect model transferability and accuracy.

An alternative to predicting atom-centered point charges has recently been used in two studies reported by Zhang et al.,^73,74^ where electrostatics are calculated using the concept of maximally localized Wannier functions.^75^ Their approach maintains the high-level form of eq 6 but expands the *U*_es_ term as a function of Wannier centers (WCs) with a charge value of 2e- and ions having a charge value of atomic nuclei + core electrons. As an example, a water molecule has four WCs each carrying 2e- and three nuclear positions with 1e, 1e, and 6e for the hydrogens and oxygen, respectively. The total system energy is calculated by representing the potential energy of WC and nuclei-centered charges with Gaussian charge distributions (*U*_G_t__).
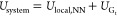
8

The calculation of *U*_G_t__ can be carried out in Fourier space as
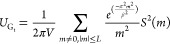
9where *L* is the Fourier space cutoff, *V* is the volume of the simulation cell, and *S*(*m*) is the charge weighted structure factor of the nuclei and WC positions. Model implementation requires two ML components to be trained using input from the local atomic environments: (1) to calculate *U*_local,NN_ and (2) to provide the WC positions. This framework has not yet been applied to a diversity of systems with nonlocal effects, but the current design effectively captures long-range electrostatic interactions, which, for example, have been demonstrated in different types of water dissociation curves. Regardless, it should be emphasized that the concept of using WCs as the basis for electrostatics is attractive because they are not restricted to the spherical simplifications of atom-centered point charges. This may lead to better MLIP predictions because of a flexible representation of the electron density distribution.

### 3.3 Models Based on Iterative Refinement

In contrast to single-step predictions of electrostatic contributions, a number of MLIP models have devised schemes based on iterative convergence. We choose to divide these models into self-consistency and message-passing methods. The defining feature of self-consistent approaches is the iterative refinement toward a converged prediction from an initial estimate, where the objective value is related to an output which itself is used to derive new inputs (for example, charges). Differing from the cyclic convergence of a self-consistent procedure, message-passing methods use consecutive calculation steps that are meant to propagate chemical information across a system through communication between neighboring atoms. These propagation steps, i.e., message passes, can be thought of as increasing the amount of nonlocal details available at each atomic site that can be used to predict chemical properties such as atomic charges. Additional message-passing steps alleviate the difficulties associated with methods only using truncated local environments, e.g., those discussed in Section 2, because input representations are refined using information about distant chemical structures that is acquired through communication with intermediate neighboring atoms. This iterative propagation of information is related to the idea of molecular graph-like representations, where atoms (nodes) are connected to their neighbors (edges) to form a “message-passing network”. One of the greatest advantages of MLIP models that use message passing is their ability to learn their own flexible chemical representations that are not bound by nearsighted features, which conceptually encourages extensibility and generalizability. Several diverse MLIP models applying these strategies are highlighted herein.

The work by Gao and Remsing reports a long-range MLIP approach called self-consistent field neural network (SCFNN),^76^ which combines an iterative refinement approach with maximally localized Wannier centers (WCs) for calculating electrostatics. The strategy of SCFNN is to execute two MLIP components sequentially: (1) a set of neural networks for predicting WC positions and the change in those positions due to an effective electric field, and (2) a set of networks for predicting local configurational and effective field forces on the atoms. A self-consistency procedure is implemented to converge the WC positions. Following initial WC estimates, via a neural network, a loop is carried out where perturbations to the WC positions are calculated based on the effective electric field, which itself is a function of the WC positions. After applying this perturbation, a new effective field is calculated, and the procedure repeats until the inferred perturbation to the WC position is below a chosen threshold (∼10^–4^ Å). Eventually a converged electric field is obtained, and the authors use it as input alongside AEV symmetry functions to calculate atomic forces with the second set of neural networks. SCFNN has been applied to a single chemical system, water, where structural features, long-range polarization, and electronic properties were reproduced with appreciable accuracy. This procedure by Gao and Remsing has some resemblance with the works of Zhang et al.;^73,74^ however, the separation of short- and long-range electrostatics, use of neural networks instead of explicit functional forms, and assumption of linear response are notable distinguishing features. Both studies report quality results for water systems, and the extent to which the approaches are generalizable to diverse systems is worth further investigation.

Xie et al. reported the so-called Becke Population Neural Network (BpopNN),^77^ which focuses on refining *q* predictions obtained from modified smooth overlap of atomic position descriptors. An innovative feature of BpopNN is the use of a self-consistent charge update scheme (SCF-q). The methodology seeks to train a ML model to learn a potential energy functional form (*U*_BpopNN_) that depends on the nuclear charges (*Z*), Becke populations (*P*), and the atomic positions (*r*).
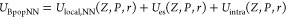
10

**Figure 4:**
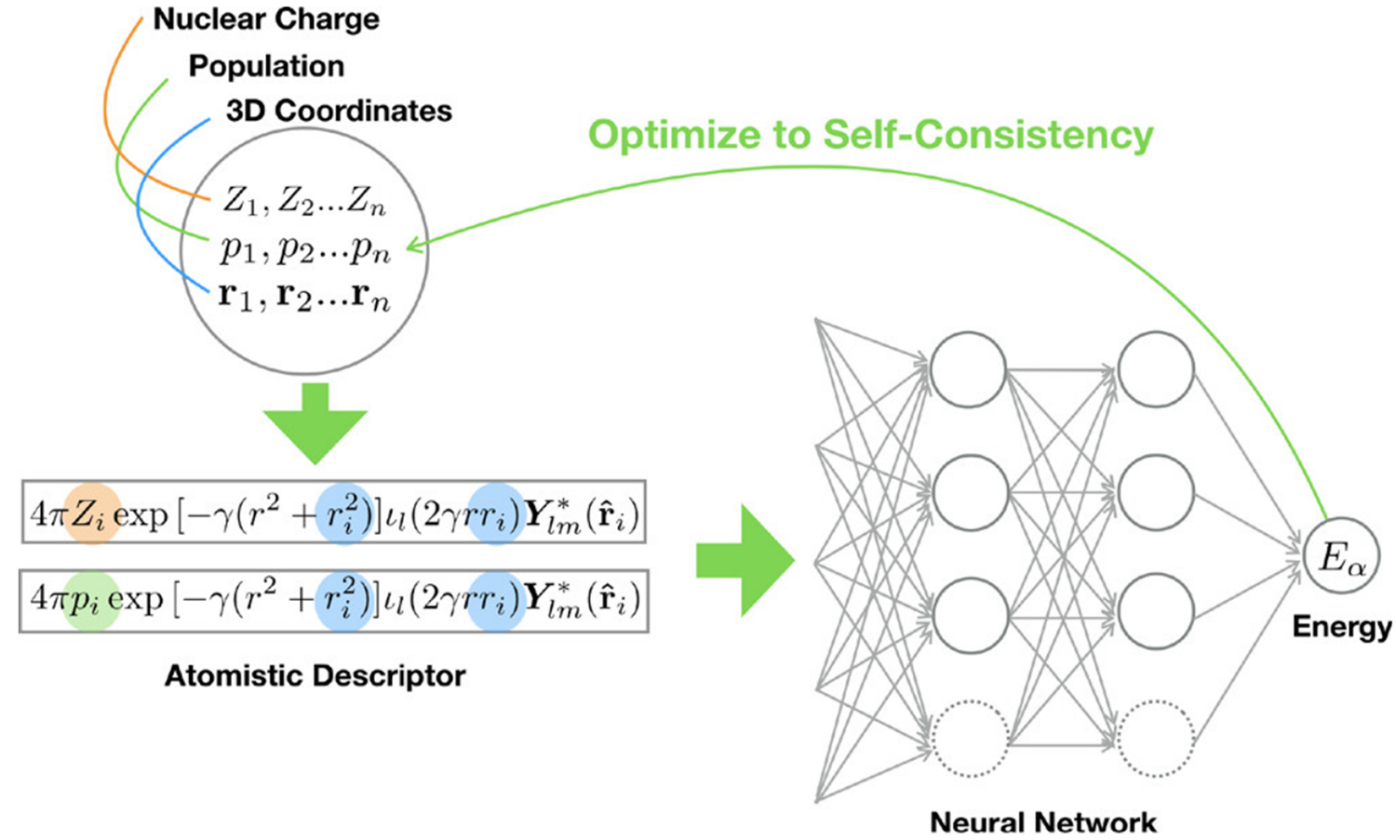
Overview of the BpopNN architecture. A self-consistent charge optimization scheme is used to iteratively refine atom-centered partial point charges and minimize the total predicted energy with respect to the overall charge distribution. Reprinted with permission from ref (77). Copyright 2020, American Chemical Society.

The potential energy contributions having a dependency on *P* enable the SCF-q scheme to be used. Following model training on constrained DFT reference data,^78^ inference can be carried out by minimizing the total energy with respect to the Becke populations , which can be performed using optimizers implemented in automatic differentiation packages, e.g., stochastic gradient descent or the Adaptive moment solver (Adam).^79^ In practice, initial guess charges are supplied, and iterative optimization steps are used to arrive at an accurate distribution of atom-centered charges. The selection of initial guess charges is a point worth emphasizing because they can influence the optimized charge distribution identified by the self-consistency procedure. An overview of the BpopNN model workflow and architecture is provided in Figure 4. To the best of our knowledge, BpopNN has only been applied to proof-of-concept examples of interaction energies, structural transitions, and charge distributions of lithium hydride systems.

Message-passing neural networks (MPNNs) are a versatile class of machine learning models that use iterative updates as part of their predictions.^80^ Each iteration, referred to as a message-passing step (*t*), increases the amount of nonlocal information available to every atom in a system. While a clear practicality issue exists with performing large numbers of message passes, for many organic systems (those without large nonlocal effects) *t* = 3 can be sufficient to reach accurate predictions.^81^ It worth expanding upon the general message-passing framework to present the mechanism that MPNN-based MLIPs can use to include nonlocal interactions. The central idea of an MLIP with message passing is for atoms in a system to maintain an abstract hidden state representation (*h*_*i*_^*t*^) that is updated through communication with nearby neighbors. The details of *h*_*i*_^*t*^ are not informed by the model developer, and the MLIP learns them during training. In most cases, this necessitates the training of two neural networks: a message-passing function and a hidden state update function. Neural networks are not strictly required, and any sufficiently flexible and learnable model can be used. Regardless, the updated hidden state maintained by atom *i* (*h*_*i*_^*t*+1^) is determined using the update function (*U*_*t*_), which transforms the previous hidden state (*h*_*i*_^*t*^) based on the cumulative message (*m*_*i*_^*t*+1^) received.
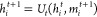
11

The message-passing function (*M*_*t*_) is responsible for defining the contribution that each neighbor of atom *i*, denoted as *N*(*i*), makes to the cumulative message. The atomic neighbors are defined up to a cutoff distance, e.g., atomic separations ≤5.0 Å.
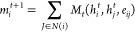
12

*M*_*t*_ is learned through training—similar to *U*_*t*_—and is a function of each atom’s hidden state and any additional features (*e*_*ij*_) between atoms *i* and *j*. The fact that eq 12 has a dependency on both *h*_*i*_^*t*^ and *h*_*j*_^*t*^ is crucial because it supports that every message-passing iteration propagates system information from further away atoms. Thus, increasingly nonlocal features can be incorporated via more message-passing steps to refine each atom’s hidden state. After a number of message-passing iterations, the hidden states can be utilized for the inference of atomic properties, including energies, charges, electronegativity, and so on.

Several models have been based on iterative update/message-passing schemes, including Deep Tensor Neural Networks (DTNNs),^82^ SchNet,^83^ SpookyNet,^84^ and AIMNet.^81^ To demonstrate the transferability of using iterative schemes, we expand on the AIMNet model, which was reported by Zubatyuk et al. in 2019 to have applicability to neutral organic molecules composed of HCNOFSCl.^81^ In AIMNet the hidden state representation takes the form of a 16-dimensional embedding vector (*a*_*z*_) that is used for defining atomic features. As an aside, the atomic embedding strategy is used because it is a solution to the unfavorable scaling problem that species-specific networks face when the number of elements in an application space grows. Regardless, the radial and angular atomic environments, as defined in Section 2, are multiplied with the atomic embeddings according to
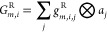
13and
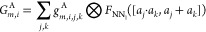
14where *g*_*m,i,j*_^R^ and *g*_*m,i,j,k*_^A^ are vectors of the *j* and *j,k* neighboring atom components of eqs 3 and 4, respectively. In eq 14, *F*_NN_ is a neural network trained to construct an effective angular embedding vector, and subscripts *i*, *j*, and *k* are the atoms composing the molecular geometry component. The *a*_*z*_ values are updated according to the scheme described above, where neighboring messages are directly accounted for up to ∼5 Å with each iterative step. The final atomic feature vector (*f*_*i*_) used for molecular property prediction is a concatenation of the terms from eqs 13 and 14 that are flattened and undergo a nonlinear transformation via another neural network.
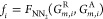
15

**Figure 5:**
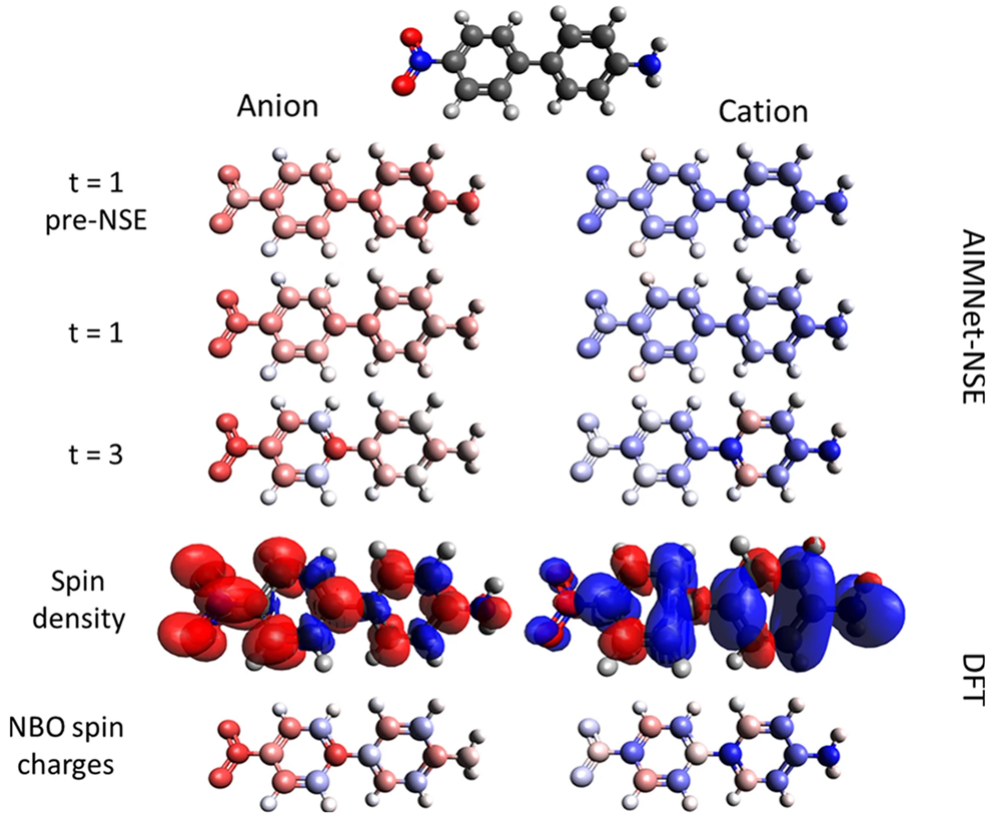
Example of using AIMNet with neutral spin equilibration and message passing to accurately assign atom-centered partial spin charges of anionic and cationic 4-amino-4′-nitrobiphenyl. Each message pass (*t*) refines the spin-charge density distribution, resulting in accurate predictions in comparison with NBO analysis. Reprinted with permission from ref (85). Copyright 2021, The Authors.

AIMNet uses *f*_*i*_ to predict system features, such as atomic partial charges, which accurately reproduce those derived from DFT calculations. Furthermore, additional nonlocal details are beginning to be included in message-passing models. For example, SpookyNet^84^ and AIMNet-NSE^85^ can both model spin states, which are valuable for simulations including reactive events and open-shell species. These models are motivated by the fact that MLIPs that rely only on nuclear degrees of freedom lack an ability to accurately infer interaction energies of species other than neutral singlets. There are a number of cases such as bond-breaking or transition-state chemistry, where failing to account for a proper electronic spin state results in a drastic misrepresentation of the potential energy surface; for example, see Figure 4b of ref (84). To highlight one working mechanism, AIMNet-NSE introduced a neutral spin equilibration procedure that is applied as
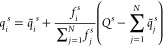
16where *Q*^s^ is the total molecular spin charge state, and *q̃*_*i*_^*s*^ and *f*_*i*_^*s*^ are partial spin-polarized atomic charges and weight factors predicted by the neural network, respectively. Subsequent message-passing steps further improve the AIMNet-NSE model prediction of localized spin charge states. As an example, Figure 5 shows spin charge state predictions of the anionic and cationic forms of 4-amino-4′-nitrobiphenyl as a function of message passes. It can be seen that, for *t* = 1, i.e., without neutral spin equilibration, the spin charge-densities are approximately equivalent. However, after *t* = 3 more accurate wave-like spin charge density distributions are predicted, showing improved agreement with DFT natural bond orbital (NBO) analysis. Overall, the ability of AIMNet-NSE to cover an application space of neutral, cationic, and anionic species with accurate spin charge density distributions is a significant step toward MLIPs with broader generalizability.

### 3.4 Potentials Incorporating Charge Equilibration

The empirical interatomic potential simulation community has been interested in calculating geometry-dependent atomic charges for several decades. For instance, force fields such as ReaxFF^86^ have accomplished this task by using charge equilibration (Qeq) schemes. For the purpose of clearly distinguishing MLIP methodologies in this Perspective, we chose to narrowly define Qeq to refer to methods that determine the charge distribution of a given configuration by solving a system of linear equations that include the interaction energies between charge densities, the atomic partial charges, and the atomic electronegativities. An important feature of this formulation is that the electrostatic interactions between all pairs of atoms are typically used to construct the charge–charge interaction energy tensor, and thus, solving the Qeq linear equations results in a fully global charge redistribution. Among the most notable Qeq methodologies proposed is the one by Rappe and Goddard,^87^ which has found recent use in MLIP formulations. The fact that Qeq methods are well-established molecular simulation techniques and are not directly restricted by nearsighted approximations makes them an attractive option to be utilized by MLIP practitioners. It should be noted that many of the MLIPs discussed below are methodologically consistent with that of Rappe and Goddard, but several charge equilibration variants exist, such as the electronegativity-equalization method (EEM),^88^ split-charge equilibration (SQE),^89^ and atom-condensed Kohn–Sham DFT approximated to second order (ACKS2),^90^ to name a few.

The Qeq approach is described with the following functional form
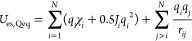
17where *χ*_*i*_ and *J*_*i*_ are the electronegativity and atomic hardness of atom *i*, respectively. The Coulomb calculation can be carried out using a long-range summation technique, e.g., Ewald or Wolf summation,^91,92^ but it has been omitted from eq 17 for clarity. The objective of Qeq is to use optimization techniques to solve the charge distribution {*q*_*i*_... *q*_*N*_} that minimizes *U*_es,Qeq_. This optimization is performed with the constraint of
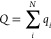
18which ensures that the sum of partial atomic charges is equal to the net charge of the system (*Q*). The distinguishing feature between MLIPs that employ Qeq formalisms for their electrostatic calculations is their treatment of *χ*_*i*_ and *J*_*i*_. A straightforward strategy is to apply parameters from existing empirical data sets. This was employed by Yoo et al.^93^ in developing a CHNO reactive MLIP, where they used *χ*_*i*_ and *J*_*i*_ from ReaxFF to model small molecule bond dissociation and various chemical features and reaction properties of 1,3,5-trinitroperhydro-1,3,5-triazine (RDX).

Instead of adopting predetermined Qeq parameters, the work of Nokikov and Shapeev applied regression techniques to derive system-specific values for silica.^94^ Their study used moment tensor potentials augmented with a standard Qeq approach to evaluate phonon spectra, structural properties, and the elastic tensor of α-quartz. Interestingly, they found the Qeq scheme with parameters obtained via iterative optimization yield no significant increase in accuracy but contributed further to prediction uncertainty. The idea of a simple combination between short-range MLIPs and charge equilibration is compelling, but this result indicates a more intimate connection between local and long-range model components might be required for predictive accuracy.

To highlight an MLIP that utilizes charge equilibration with integrated short- and long-range components, the so-called fourth-generation Behler-Parrinello NNPs (4GNNP) of Behler, Goedecker, and co-workers^95^ is discussed. 4GNNP is a slightly modified local Behler-Parrinello model combined with the CENT architecture, which is an ML-based charge equilibration framework originally aimed at ionic crystal applications.^96^ The total energy of a 4GNNP uses the same high-level form of eq 6, where the calculation occurs in two parts. In the first step, the CENT scheme is applied to calculate *U*_es_ from the interaction of atomic charge densities. A Gaussian functional form is used to distribute the charges centered on atomic positions as
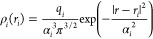
19with *r*_*i*_ and *α*_*i*_ being the atomic position and distribution width parameter, respectively. Qeq is applied to determine the solution of {*q*_*i*_... *q*_*N*_} that minimizes *U*_es,CENT_, which is expressed similarly to eq 17 as a truncated Taylor expansion in the following form
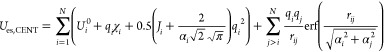
20where *U*_*i*_^0^ is an atomic reference energy. A key contribution of CENT is that *χ*_*i*_ is assumed to be environment-dependent and predicted with an ML model from an AEV input representation. It is worth highlighting that there is methodological nuance between the training procedure of the standalone CENT approach and 4GNNPs, and the interested reader is directed to refs (19 and 95) on this point. Regardless, the charge optimization process is a global system operation carried out with electronegativity parameters that vary depending on the local arrangement of atoms. This is the main mechanism that allows for the 4GNNP to account for events like nonlocal charge transfer.

**Figure 6:**
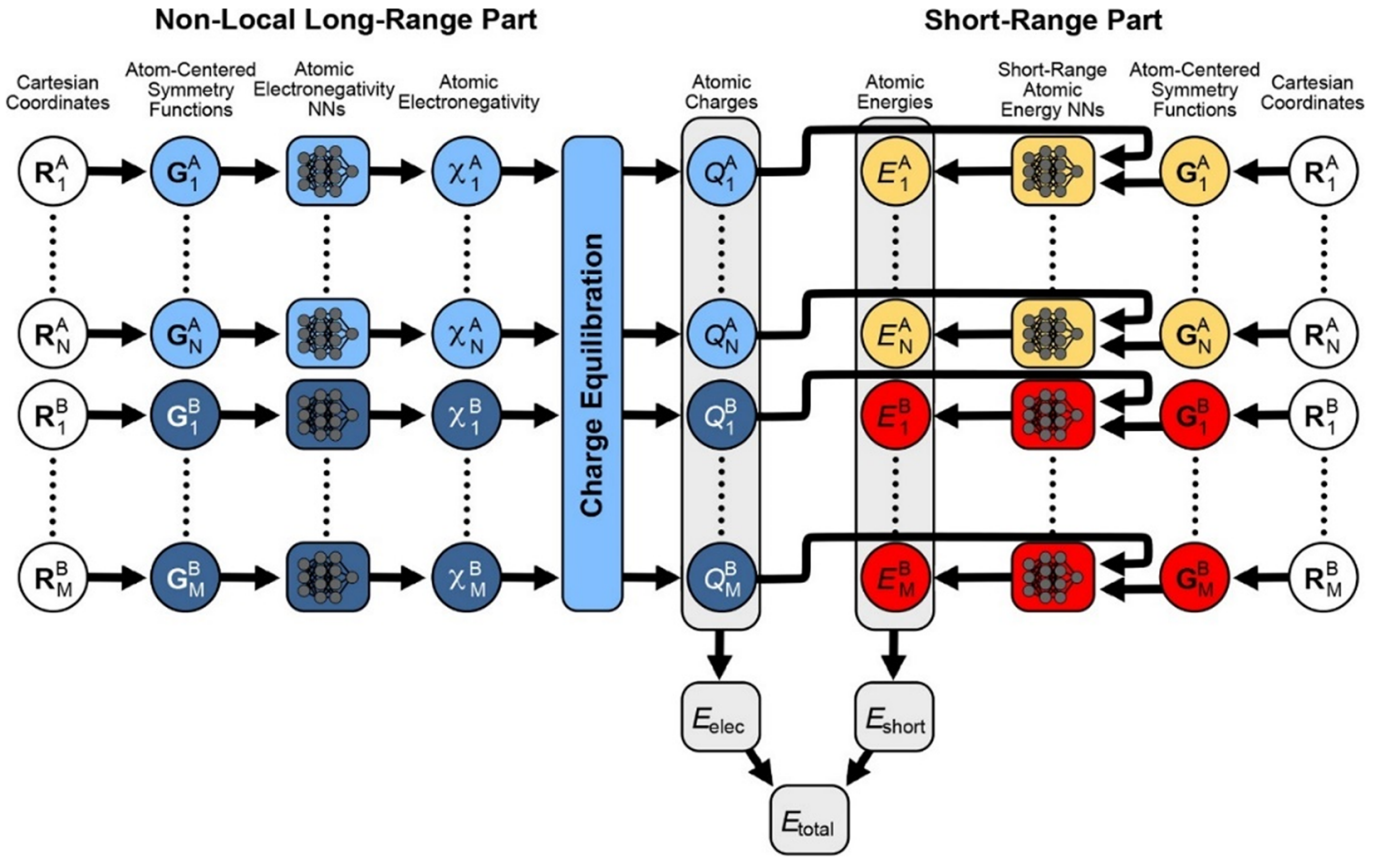
Schematic overview of the 4GNNP architecture. A charge equilibration scheme (shown on the left) is employed to obtain the globally optimized partial point charges used in calculating long-range electrostatic interactions. The short-range potential (shown on the right) involves the 2nd generation BPNNP with an additional input parameter for the charge value on each atom. Reprinted with permission from ref (97). Copyright 2021, American Chemical Society.

The short-range contribution (*U*_local,NN_) is calculated following the optimization of {*q*_*i*_... *q*_*N*_}. Consistent with the description in Section 2, the calculation *U*_local,MLIP_ in the 4GNNP framework is a summation of per atom energies predicted from element-specific neural networks. A change is made to the input feature vector to also include the charge value determined in the CENT step. Considering this charge value is obtained from a global process, it brings a unique contribution to what would otherwise be a neural network operating on strictly short-range features. An illustrative example of the 4GNNP architecture is shown in Figure 6. The prospect of the 4GNNP scheme is promising with demonstrated success for challenging systems, including ionic long linear alkanes, small sodium chloride clusters, and interactions with a magnesium oxide surface, to name a few.^95,97^

## 4 Polarization, ML/MM, and Beyond

Our discussion up to this point has mainly focused on MLIPs using pairwise dispersion, some comments on the importance of many-body effects, and electrostatics via Coulomb’s law (predominately via point charges). Nevertheless, it is known that intermolecular interactions that extend beyond local atomic environments consist of electrostatics, dispersion, and induction;^98^ therefore, MLIP models neglecting one or more of these terms are incomplete by design. Most MLIPs discussed in the previous sections that make such assumptions are either applied pragmatically or they do so with a justifiable basis from a chemical understanding of the target application space. Regardless, long-range interactions between molecules and materials are defined by the electric fields produced by the electron density distribution of each species, and thus, electrostatics are accurately described using a multipole expansion. Long-range interactions are often coupled, and neighboring molecules and materials produce deformations of electron densities from the isolated reference state (i.e., polarization) with an associated induction energy. Therefore, an MLIP model that aims to accurately represent an application space where polarization effects have significant contributions beyond *U*_local,MLIP_ must include explicit functional forms or features operating on the length scales of relevant electron density redistribution. One of the simplest approximations of polarization effects in an MLIP is an electrostatic potential calculated via partial atomic charges that vary as a function of atomic environments. As we have previously noted, reducing a complex electron density distribution to atom-centered point charges can produce non-negligible error,^99^ and as a result, this is a poor approximation to account for the induction energy of a system with long-range polarization.

To the best of our knowledge, the only model that includes a dedicated mechanism for long-range polarization contributions is the previously discussed report by Gao and Remsing (see Section 3.3). Their approach operates through self-consistent determination of Wannier center locations through linear perturbations in response to the effective electric fields at these positions. Gao and Remsing’s SCFNN model was shown to accurately predict the high-frequency dielectric constant of water, which demonstrates reliable long-range electrostatic screening effects. These results are encouraging; however, the SCFNN model relies on linear response and the partitioning of DFT data into short- and long-range contributions. It is worth commenting that these dependencies do not limit Gao and Remsing’s study of chemically homogeneous water systems, but systematically extending this framework to a broad range of chemistry does not appear straightforward. We are unaware of any other MLIP that includes a dedicated explicit mechanism for incorporating induction energy beyond local features. Momentarily disregarding the issues associated with partial point charges (i.e., monopole electrostatics), it could be argued that charge equilibration or message-passing methods can incorporate longer-range charge transfer effects and are better suited for polarizable systems. While this is partially accurate, these methods also have limitations arising from local approximations. For message-passing MLIPs, such as AIMNet, intermediate particles are required to allow the update function to propagate information about nonlocal structure. Thus, a polarizing body whose nearest neighbor is more than one AEV cutoff distance away is effectively treated as an independent system from the message-passing perspective. This is less of an issue for condensed phases, but it can cause incorrectly predicted physics in low-density systems (e.g., vapors). Turning to the Qeq methods, these are performed as a global system operation that allows charge to redistribute by minimizing the electrostatic energy via the distribution of partial charges, which can conceivably capture a degree of polarization effects. For a specific example, the aforementioned 4GNNP predicts electronegativities from local features before carrying out Qeq; however, these are chemically ambiguous properties that also suffer from truncated AEVs, and a similar limitation exists for describing long-range polarization contributions. It is potentially worthwhile to consider strategies used in classical molecular simulations to provide inspiration for MLIP models designed for long-range polarizable systems. A frequently applied empirical model that is based on a multipole expansion is the AMOEBA force field, where permanent atomic multipoles (up to the quadrupole) and polarizable dipoles are used to calculate electrostatic interactions.^100,101^ Adapting this representation to an MLIP framework could be valuable for modeling polarizable systems. Briefly, in addition to interactions between permanent electrostatic multipoles, AMOEBA explicitly includes many-body polarization as
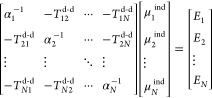
21, where α, *T*^d-d^, μ^ind^, and *E* are the atomic polarizability tensors (usually isotropic), Thole damped interaction tensors,^102^ induced dipoles, and the polarization electric field at each site, respectively.

An important application where polarization effects can dictate system properties is the so-called machine learning/molecular mechanics (ML/MM) method (e.g., refs (103−105)), which is related to the quantum mechanics/molecular mechanics (QM/MM) approach presented in a landmark work by Warshel and Levitt.^106^ For an example of ML/MM, Inizan et al. recently applied ANI MLIPs to calculate chemically accurate solute–solute interactions, whereas solute–solvent and solvent–solvent interactions were simulated with AMEOBA.^107^ In ML/MM, inference is affected by the electric field induced by the molecular mechanics region, and therefore, training beyond unperturbed gas-phase QM data is required in the absence of explicit long-range features. The challenge can compound when mutual polarization is considered; i.e., the MM components are described using a classical polarizable force field, such as AMOEBA.^108^ The difficulty of simulating these systems may eventually be overcome by methods using only MLIPs with high-fidelity long-range electrostatics/polarization, i.e., ultimately replacing the MM region, need for embedding schemes, and complicated Hamiltonian formulations. A strategy to perform ML/MM simulations for highly polar systems with a mechanical embedding scheme is to train an MLIP model to infer atomic contributions to the multipole moments. As an example, reports by Poplier and co-workers have demonstrated the use of data-driven techniques,^109−111^ particularly Gaussian process regression, to infer atomic electrostatic moments derived in the framework of the quantum theory of atoms-in-molecules.^112^ The challenge of developing MLIPs with accurate polarization and electrostatic multipoles is ongoing, but a number of works are starting to address these issues.^70,113−115^

## 5 Outlook and Concluding Remarks

MLIPs are reaching a mature status, and their use in molecular simulations is becoming a frequented method in the computational chemist’s toolbox. This Perspective highlighted specific models and methods that are expanding the application space of MLIPs beyond systems with structure/properties dominated by short-range physics and chemistry. We emphasized that the approximation of atomic interaction locality can constrain the variety of systems an MLIP can simulate. To overcome this, models that capture interactions and environment changes beyond an atom’s immediate vicinity have been reported, indicating that MLIP-driven simulations for bulk, biological, and material systems are emerging areas.

Debatably, the bottleneck to increasing MLIP capabilities is the accumulation of reference data for training, instead of limitations resulting from the underlying model architecture. It is currently unclear the amount and diversity of system sampling required to train MLIPs with reliable long-range interactions. The issue of limited data is persistent across ML fields, and active learning is one strategy that can address this challenge.^116^ In the paradigm of active learning, redundant training practices are reduced by maximizing training data diversity and limiting data set size such that each sample meaningfully contributes to refining model parameters. For instance, an active learning strategy known as query by committee was used in producing the ANI-1x model,^117^ where less than 25% of the original ANI-1 training data was needed to achieve equal accuracy.^118^ Active learning strategies are key for building the next generation of MLIPs considering that complex models often require more training data. Moreover, MLIP accuracy and transferability are dependent on the composition of the reference data set. The development of methods that minimize the training size and maximize the value of each data point is essential to build MLIPs for practical use. Although not reported, there is envisioned value in systematic studies that establish “rule of thumb” design principals^119^ between accuracy, MLIP architecture with long-range interactions, and active learning strategies.

The emergence of MLIPs with nonlocal interactions creates opportunities for insight into complex systems with exceptional accuracy. A shift in research focus from “improving accuracy on standard benchmarks” to performing simulations aimed at measurable scientific progress is timely and encouraged. The practice of judging MLIP models against each other will continue in all likelihood; however, we advocate for these future comparisons to occur on sets of standard benchmarks of experimental ground truths. Unfortunately, such experimental data sets either do not exist or are not yet widely embraced by MLIP developers, and efforts to curate these, particularly for noncovalent interactions, is a worthwhile pursuit. By our assessment, the number of different MLIP models greatly exceeds the cases in which they have been uniquely successful for understanding a chemical or materials science challenge. Most MLIPs have only been tested on a handful of systems in simple trial studies, and as a consequence, the area of applied MLIP modeling lags behind model development. Exploring new ML methods and developing complex model architectures are appreciable pursuits; however, it is worthwhile to interrogate such efforts for the value they provide beyond existing MLIPs. We emphasize that MLIPs are subject to an adage of classical simulations: a model should be no more or no less complex than what the application space demands. In the context of this Perspective, the complexity involved in treating long-range interactions should be judicious, for example, based on rational physical and chemical knowledge. MLIP models should be designed with treatments of electrostatics, polarization, dispersion, and many-body effects that coincide with relevant material or chemical phenomena that dictate the properties or structures of interest. On one hand, the treatment of long-range interactions in the design of an MLIP can be intuited by the molecular simulation practitioner: we have highlighted examples in Section 3.1 where it is possible to rationalize only using *U*_local,MLIP_ and explicit dispersion correction because the condensed-phase systems were neutral and chemically homogeneous. On the other hand, some tasks may require using a MLIP in a bulk phase application setting to understand its deficits and incorporate refinements. Such cases further support the importance of moving MLIP development beyond benchmarks and proof-of-concept studies.

Many reports now exist where nearsighted MLIPs have been applied to simulate large-scale systems (>10^7^ atoms), for example, Smith et al. for aluminum,^120^ Guo et al. for copper,^121^ and Lu et al. for water and copper.^122^ With the advent of exascale computing,^123^ it is expected that simulations for similar system sizes using MLIPs with long-range interactions will soon follow. MLIP-based simulations on this scale can yield unprecedented understanding into challenges faced across physics, chemistry, and engineering disciplines.^124^ However, a remaining limitation to deploying MLIPs for such efforts is efficient integration with software capable of performing molecular simulations, particularly molecular dynamics and Monte Carlo methods. Examples of MLIP implementations into existing molecular simulation software are the DeePMD-kit,^125^ AENET,^126^ SNAP,^28^ and TorchANI.^127^ Despite these successes, there are a number of long-range MLIP models, such as those using message passing or Qeq, that are challenging to efficiently plug into existing software, and further development effort is needed. There is also appreciable optimization space available at the hardware and algorithm levels, for example, see the works of Guo et al.^121^ and Galvelis et al.^128^ Constructing MLIP interfaces with simulation packages that fully leverage the power of accelerated computing architectures is a nontrivial task. Unified effort between MLIP development, model implementation, and performance optimization is an ongoing need.

This year marks nearly a decade and a half of high-dimensional MLIP research, during which models have progressed to cover a span of short-ranged to nonlocal physics and chemistry. Even though broadly generalizable models for condensed matter with long-range interactions are in an infantile state, system/species specific MLIPs are positioned to revolutionize computational chemistry and materials science fields. Many challenging areas remain where molecular simulations using MLIPs with long-range interactions have yet to experience widespread success. Condensed-phase chemical reactions, interactions with light, and complicated catalytic events are a few areas in the chemical sciences that can benefit from MLIP model development. For materials science, magnetic materials, complex behavior of defects, and composite interfaces are modestly explored areas where MLIP models with long-range interactions can be transformative. The field of MLIPs has experienced rapid progression, and the application space continues to expand. Thus, we envision simulating systems with complex short- and long-range behavior using accurate MLIPs will be the basis of many future scientific breakthroughs.
